# Chrysin-loaded PEGylated liposomes protect against alloxan-induced diabetic neuropathy in rats: the interplay between endoplasmic reticulum stress and autophagy

**DOI:** 10.1186/s40659-024-00521-1

**Published:** 2024-07-09

**Authors:** Mahran Mohamed Abd El-Emam, Amany Behairy, Mahmoud Mostafa, Tarek khamis, Noura M. S. Osman, Amira Ebrahim Alsemeh, Mohamed Fouad Mansour

**Affiliations:** 1 Department of Biochemistry and Molecular Biology, Zagazig University, Zagazig, 44511 Egypt; 2 Department of Physiology, Zagazig University, Zagazig, 44511 Egypt; 3 Department of Pharmaceutics, Faculty of Pharmacy, Minia University, Minia, 61519 Egypt; 4 Department of Pharmacology, Faculty of Veterinary Medicine, Zagazig University, Zagazig, 44519 Egypt; 5 Laboratory of Biotechnology, Faculty of Veterinary Medicine, Zagazig University, Zagazig, 44519 Egypt; 6 Department of Human Anatomy and Embryology, Faculty of Medicine, Port Said University, Port Said, Egypt; 7 Human Anatomy and Embryology Department, Faculty of Medicine, Zagazig University Egypt, Zagazig, Egypt

**Keywords:** Alloxan-induced diabetes, Neuropathy, Chrysin PEGylated liposomes, Endoplasmic reticulum stress, Autophagy

## Abstract

**Background:**

Diabetic neuropathy (DN) is recognized as a significant complication arising from diabetes mellitus (DM). Pathogenesis of DN is accelerated by endoplasmic reticulum (ER) stress, which inhibits autophagy and contributes to disease progression. Autophagy is a highly conserved mechanism crucial in mitigating cell death induced by ER stress. Chrysin, a naturally occurring flavonoid, can be found abundantly in honey, propolis, and various plant extracts. Despite possessing advantageous attributes such as being an antioxidant, anti-allergic, anti-inflammatory, anti-fibrotic, and anticancer agent, chrysin exhibits limited bioavailability. The current study aimed to produce a more bioavailable form of chrysin and discover how administering chrysin could alter the neuropathy induced by Alloxan in male rats.

**Methods:**

Chrysin was formulated using PEGylated liposomes to boost its bioavailability and formulation. Chrysin PEGylated liposomes (Chr-PLs) were characterized for particle size diameter, zeta potential, polydispersity index, transmission electron microscopy, and in vitro drug release. Rats were divided into four groups: control, Alloxan, metformin, and Chr-PLs. In order to determine Chr- PLs’ antidiabetic activity and, by extension, its capacity to ameliorate DN, several experiments were carried out. These included measuring acetylcholinesterase, fasting blood glucose, insulin, genes dependent on autophagy or stress in the endoplasmic reticulum, and histopathological analysis.

**Results:**

According to the results, the prepared Chr-PLs exhibited an average particle size of approximately 134 nm. They displayed even distribution of particle sizes. The maximum entrapment efficiency of 90.48 ± 7.75% was achieved. Chr-PLs effectively decreased blood glucose levels by 67.7% and elevated serum acetylcholinesterase levels by 40% compared to diabetic rats. Additionally, Chr-PLs suppressed the expression of ER stress-related genes (ATF-6, CHOP, XBP-1, BiP, JNK, PI3K, Akt, and mTOR by 33%, 39.5%, 32.2%, 44.4%, 40.4%, 39.2%, 39%, and 35.9%, respectively). They also upregulated the miR*-*301a*-*5p expression levels by 513% and downregulated miR*-*301a*-*5p expression levels by 65%. They also boosted the expression of autophagic markers (AMPK, ULK1, Beclin 1, and LC3-II by 90.3%, 181%, 109%, and 78%, respectively) in the sciatic nerve. The histopathological analysis also showed that Chr-PLs inhibited sciatic nerve degeneration.

**Conclusion:**

The findings suggest that Chr-PLs may be helpful in the protection against DN via regulation of ER stress and autophagy.

**Graphical Abstract:**

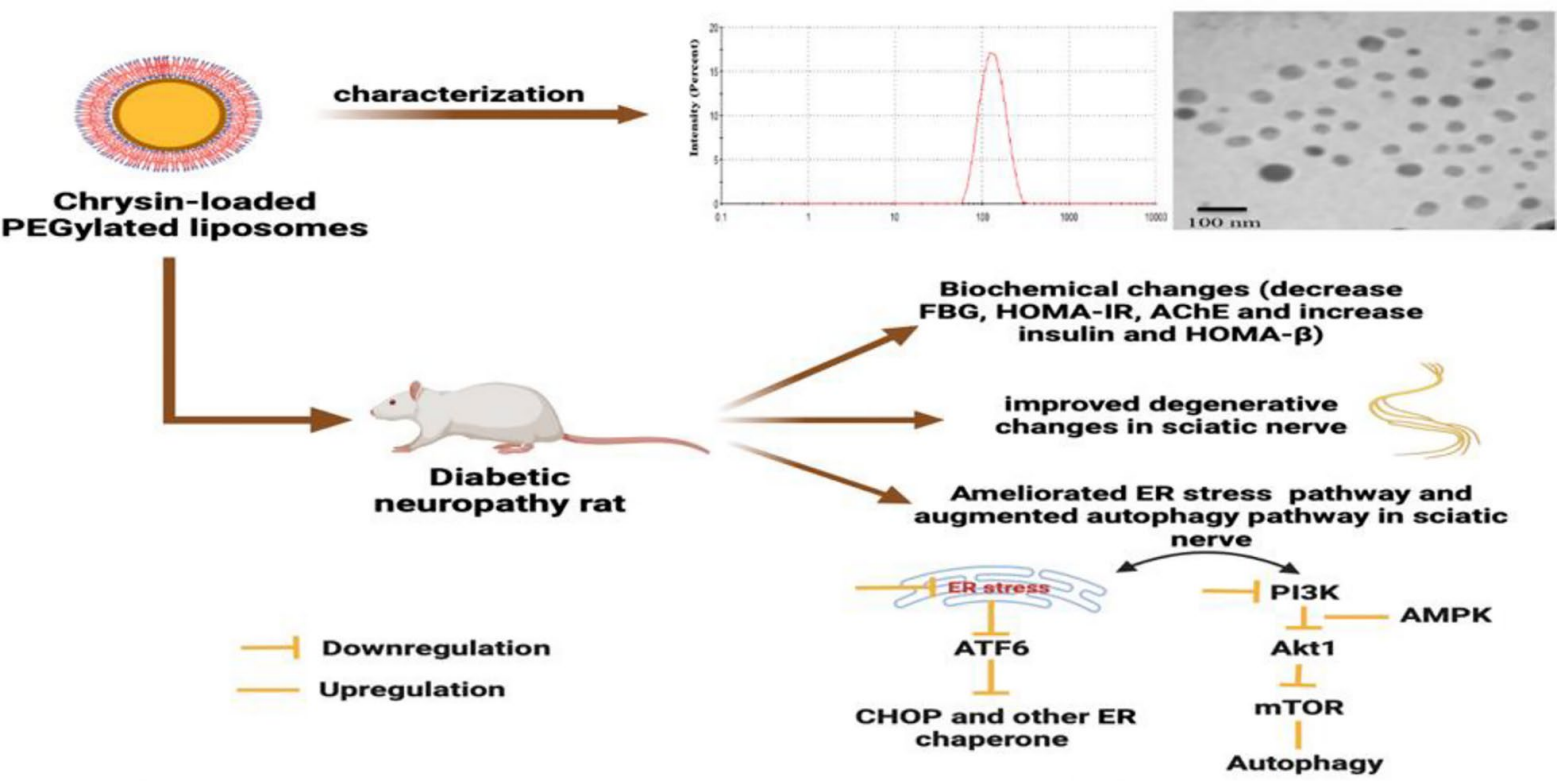

## Background

Diabetes mellitus (DM) refers to a group of metabolic disorders characterized by high glucose levels in the blood and insufficient production or effectiveness of insulin by the pancreas [1]. According to the World Health Organization, the global population affected by diabetes currently exceeds 400 million individuals. However, this number is projected to rise significantly and reach 552 million by 2030 [2]. Diabetic neuropathy (DN), which includes allodynia, hyperalgesia, and spontaneous pain, is a severe complication of the disease. About half to two-thirds of people with diabetes would develop diabetic neuropathy [3]. Controlling DN entails mostly maintaining normal blood sugar levels and treating symptoms [4]. There has been limited progress in alleviating DN-related chronic pain since undesirable side effects accompany many pharmacological medications, and therapeutic expectations remain largely unmet [5]. So, it is crucial to develop new therapy strategies for DN.

Endoplasmic reticulum (ER) stress may contribute significantly to DN’s development. ER stress is distinguished by the buildup of imperfect proteins within the ER stress, which can hinder its ability to fold proteins properly [6]. Unfolded protein response (UPR), which regulates translation, enhances protein folding, and affects inflammation in abnormal conditions, is triggered by ER stress in cells [7]. UPR is initiated by three canonical UPR mediators (sensors), including inositol-requiring enzyme 1α (IRE1α), protein kinase R-like ER kinase (PERK), and activating transcription factor 6 (ATF6) pathways [8]. These mediators attach to binding immunoglobulin protein (BiP) in an inactive state. Stress causes BiP to separate from the ER and aid in protein folding, activating PERK and ATF6 sensors [9]. IRE1α, an evolutionarily conserved ER stress sensor, initiates X-box-binding protein 1’s unconventional mRNA splicing, resulting in XBP1s, an active transcription factor that enhances the ER’s capacity to handle and remove unfolded proteins [10]. IRE1 mediates ER stress by promoting the phosphorylation of C-jun NH2-terminal kinase (JNK). PERK, a key factor in the UPR response, is activated by phosphorylating the α-subunit of eIF2α, which hinders the assembly of the 80 S ribosome and protein synthesis [11]. Additionally, under ER stress, ATF6 exports to the Golgi apparatus, cleaves at Sites 1 and 2, and translocates to the nucleus for transcription of UPR target genes, including CCAAT-enhancer-binding protein homologous protein (CHOP) [12]. Conversely, continuous activation of ER stress leads to cellular breakdown, such as in the case of high blood sugar levels.

Autophagy is a natural response to stress that assists in the breakdown of pathogens, denatured proteins, and impaired organelles within the lysosomes [13]. There was a robust mechanistic association between ER stress and autophagy [14]. JNK has been connected to IRE1 activation, which could result in autophagy caused by Beclin-1 [15]. In addition, during ER stress, autophagy activation is dependent on PERK-eIF-2, indicating a connection between autophagy and the UPR signalling pathway. Mammalian target of rapamycin (mTOR) prevents AMP-activated protein kinase (AMPK) from interacting with UNC-51-like kinase 1 (ULK1), which in turn prevents autophagy [16]. The PI3K/AKT/mTOR pathway is primarily studied for its regulation of autophagy, involving PI3K, AKT, and mTOR as key molecules. PI3K is a cytoplasmic lipid kinase that can phosphorylate phosphatidylinositol at the D3 position [17]. The regulatory component maintains the catalytic subunit in a low-activity state during physiological circumstances. External stress triggers the phosphorylation of the SH2 domain of the p85, releasing the restriction on the p110 and activating PI3K and its downstream signaling pathways [18]. AKT is phosphorylated by PKC-1 and mTOR2 upon PI3K activation, transforming into p-AKT and transported to the cell membrane [19]. By transducing signals to mTOR, p-AKT inhibits autophagy physiologically by activating the ubiquitin–proteasome pathway and regulating genes associated with autophagy or other downstream substrates [20]. The PI3K/AKT/mTOR signaling pathway is involved in signal transmission, autophagosome movement, and vesicle fusion in the autophagy process [21]. Thus, regulation of the PI3K/AKT/mTOR signaling pathway is crucial for maintaining autophagy's homeostasis. Autophagy dysfunction and ER stress were both found to be associated with diabetes [22]. Interventions aiming to normalize ER stress and autophagy may be proposed as an effective means of limiting DN development.

Various researchers are now studying natural therapies for various medical issues. Chrysin (Chr) 5,7-dihydroxyflavone, is a flavonoid abundant in *passiflora, chamomile, oroxylum* and honey, and propolis. It possesses considerable antioxidant activity and various pharmacological advantages, such as antidiabetic, anti-inflammatory, and protective effects on the heart and liver [23]. Nevertheless, the inadequate bioavailability and low solubility of chrysin, which varies between 2 and 5 µg/mL, pose significant challenges to its use in therapeutics [24]. In addition, chrysin undergoes many pre-systemic metabolism, including glucuronidation and sulphation in the intestine and liver [25]. For this reason, researchers investigated potential carriers that could overcome these obstacles and enhance the bioavailability of drugs exhibiting suboptimal pharmacokinetics. Drug delivery systems based on nanotechnology are the most popular for this purpose [26].

Liposomes are lipid-based nanocarriers that bring drugs to their target sites [27]. The bioavailability of poorly soluble medications in water can be boosted by encapsulating them in liposomes [28]. Using liposomes, which are believed to be biocompatible, biodegradable, non-toxic, and immunogenic, reduces the risk of adverse drug reactions during administration [29]. Furthermore, liposomes can improve the pharmacokinetic features of a drug, providing a way to achieve the optimal concentrations required for its intended action [30]. Liposomes’ half-lives in the bloodstream can be increased from a few minutes to several hours thanks to hydrophilic polymers like PEG employed as surface coatings [31]. In addition to blocking the reticuloendothelial system from ingesting and opsonizing liposomes, PEG boosts their solubilization power, decreases their aggregation, and lowers their immunogenicity [32]. PEG lengthens the time it takes for blood to circulate and causes more liposomes to accumulate in damaged tissues [33]. This PEG technology is used in the pharmaceutical product Doxil®, which has a potent antitumor action [34].

This work hypothesized that administering Chr-PLs to rats with DN may reduce the ER stress response and promote autophagy by controlling the related genes. The influences of Chr-PLs on insulin, fasting blood glucose level (FBG), and acetylcholinesterase in diabetic rats were investigated to test the hypothesis. The effects of Chr-PLs on ER and autophagy-related markers in diabetic rats were further analyzed by measuring their mRNA, microRNAs (miRNAs), and protein expression levels. Furthermore, Chr-PLs’ influence on the histopathology of the sciatic nerve was demonstrated. The findings of this research offer a distinct perspective on managing ER and autophagy effects and the effective management of peripheral neuropathy by novel Chr-PLs.

## Materials and methods

### Chemicals

Alloxan, polyethylene glycol 4000 (PEG_4000_), and cholesterol were provided by Sigma-Aldrich (St. Louis, MO, USA). Lipoid GmbH (Ludwigshafen, Germany) supplied saturated phosphatidylcholine derived from soybean. Chrysin (99.2%) was purchased from Axenic (Oak Park, Melbourne, Australia).

### Animals

We obtained healthy adult male Sprague–Dawley rats from the experimental animal unit at the College of Veterinary Medicine at Zagazig University, Egypt. These rats weighed between 200 and 250*g*. Before the experiment, the rats had a standard commercial food and water diet. Additionally, they were given 2 weeks to acclimate to the laboratory environment, which maintained a temperature of approximately 25 °C.

### Preparation of a nano-liposomal formulation for chrysin

The solvent injection method was utilized to produce chrysin-loaded PEGylated liposomes (Chr-PLs), as described elsewhere [35, 36]. To develop the organic phase, saturated phosphatidylcholine from soybean, cholesterol, chrysin, and PEG_4000_ were added to 100% ethyl alcohol in the following proportions: 13:3:1:1 (w/w). The temperature of the solution was increased to 60–70 °C. However, an aqueous phase consisting of a 0.9% sucrose solution was maintained at the same temperature while agitated with a magnetic stirrer (1000 rpm). In order to enable the assembly of liposomes, the organic phase was infused into the aqueous phase via a 25G syringe. Following the injection procedure, the mixture was maintained at 60–70 °C for 20–30 min in order to facilitate the evaporation of ethyl alcohol [37]. Particle size diameter, polydispersity score, zeta potential, transmission electron microscope (TEM), and an in vitro release study were all used to characterize the newly generated Chr-PLs formulation. The produced liposomal suspension's particle size diameter, polydispersity score, and zeta potential were measured using the Malvern Zetasizer Nano (Malvern Instruments Ltd., Worcestershire, UK) as previously reported [35, 38]. After the liposomal systems were lysed, the entrapment efficiency of the resulting systems was assessed, as previously reported [39]. In brief, 1 ml of Chr-PLs was centrifuged at 10,000 rpm for 60 min (Hermle, Essen, Germany) and the pellet was resuspended for three times to remove the unentrapped drug. Chr-PLs were mixed with acetonitrile (1:4) and sonicated for 20 min. The amount of Chr was determined spectrophotometrically at 267 nm (Shimadzu, Kyoto, Japan) and the entrapment efficiency was calculated as follows:

$$\text{Entrapment efficiency}= \frac{\text{Amount of Chr in liposomes}}{\text{Total amount of Chr}}\times 100$$

An in vitro release study was conducted according to Abd El-Emam et al. [39]. Briefly, an aliquot of Chr-PLs was placed within the sample compartment of an established Franz diffusion cell. The release medium was composed of PBS (7.4)—ethanol mixture (65:35) which was placed into the reservoir chamber. The two chambers were separated using A nitrocellulose membrane (12–14 kDa MWCO). The system was operated at 60 rpm and 37 °C. Two milliliters of reservoir medium were obtained at regular intervals and subjected to UV examination at a wavelength of 267 nm to quantify the amount of Chr released. The volume of the reservoir medium was restored using an equivalent volume of PBS-ethanol mixture maintained at 37 °C.

### Experimental design

#### Induction of diabetes

Rats were injected with 150 mg/kg of alloxan solution intraperitoneally (i.p.) following an overnight fast to induce diabetes [40]. For 48 h, the rats were given sucrose to avoid their deaths from the rise in insulin. After 72 h, a Glucometer was used to assess glucose levels in blood samples collected from the tail veins of the rats. Only diabetic rats (defined as having a fasting blood glucose level of 250 mg/dL or more) were employed to proceed with the experiment.

##### Experimental groups

Forty rats were randomly assigned into four groups (n = 10). Control group, Alloxan group (Alloxan-treated rats), metformin group (Alloxan-treated rats received metformin orally, 100 mg/kg BWt, once a day for 21 successive days), and Chr-PLs group (5 mg/kg BWt, i.p., every other day for 21 consecutive days) [41].

Following the last treatment, the rats were fasted overnight. Body weight was recorded, and Blood samples were collected through a cardiac puncture while the rats were under diethyl ether anesthesia. Blood was drawn into tubes without an anticoagulant to separate the serum and tubes containing sodium fluoride to separate plasma. A small cross-section from sciatic nerves was promptly removed and fixed in a 10% formaldehyde solution for histopathological and immunohistological analysis.

### Biochemical analysis

Insulin levels were determined using the rat insulin ELISA technique (Catalogue Number ERINS, Thermo Fisher Scientific, Waltham, USA), while FBG levels were evaluated using the glucose oxidase method (Agape Diagnostics Ltd., Kochi, India). The blood acetylcholinesterase levels of each group were determined using a colorimetric kinetic assay (Biodiagnostic, Giza, Egypt). The HOMA-IR value was determined by employing the subsequent equation [42]:

$$\frac{{{\text{Fasting\, glucose}} \left( {\frac{{{\text{mmol}}}}{{\text{L}}}} \right) \times {\text{fasting\, insulin}} \left( {\upmu {\text{IU}}/{\text{mL}}} \right)}}{22.5}$$

while HOMA-β was determined by employing the subsequent equation[42]:

$$\frac{{360 \times {\text{fasting\, insulin}} \left( {\frac{{\text{U}}}{{{\text{ml}}}}} \right)}}{{{\text{Fasting\, plasma\, glucose}} \left( {\frac{{{\text{mg}}}}{{{\text{dl}}}}} \right){-} 63}}$$

### Real-time RT-PCR

Total RNA was extracted from the sciatic nerve sample using the TRIzolTM reagent kit following the manufacturer’s instructions (Invitrogen, Thermofisher Scientific, Waltham, MA, USA). As was previously reported, 500 ng of total RNA was used for transcription, producing mRNA [43, 44]. In this case, miRNA transcription was performed using TaqManTM Small RNA Assays (Thermofisher Scientific, Waltham, MA, USA) on ten ng of RNA according to the manufacturer’s guidelines. Primers specific to miRNAs, stem-loops, and the universal reverse primer were all designed with the help of http://genomics.dote.hu:8080/mirnadesigntool (viewed on 10 September 2020) assay design software [45]. Sangon Biotech (Beijing, China) kindly supplied a list of the primers used in this investigation (Table 1). For real-time PCR, we used the Maxima SYBR Green/Rox qPCR 2× Master Mix from Thermofisher Scientific in Waltham, MA, USA. Each gene’s relative expression was calculated using the 2^–ΔΔCt^ technique, with mRNA and miRNA normalized to housekeeping GAPDH and U6, respectively [46].

**Table 1:** primer sequences used in real time PCR

Genes	Forward primer sequence (5ʹ-3ʹ)	Forward primer sequence (5ʹ-3ʹ)	product size (bp)	Accession number
Beclin-1	GAATGGAGGGGTCTAAGGCG	CTTCCTCCTGGCTCTCTCT	180	NM_001034117.1
LC-3	GAAATGGTCACCCCACGAGT	ACACAGTTTTCCCATGCCCA	147	NM_012823.2
mTOR	GCAATGGGCACGAGTTTGTT	AGTGTGTTCACCAGGCCAAA	94	NM_019906.2
P62	GGAAGCTGAAACATGGGCAC	CCAAGGGTCCACCTGAACAA	183	NM_181550.2
AMPK	GCGTGTGAAGATCGGACACT	TGCCACTTTATGGCCTGTCA	103	NM_023991.1
AKT-1	GAAGGAGAAGGCCACAGGTC	TTCTGCAGGACACGGTTCTC	111	NM_033230.3
ULK-1	CGTACACTGCCTGACCTCTC	AGAGGCCTGTGTCCCAAATG	162	NM_001108341.1
JNK	AGTGTAGAGTGGATGCATGA	ATGTGCTTCCTGTGGTTTAC	182	NM_053829.2
CHOP	CACAAGCACCTCCCAAAG	CCTGCTCCTTCTCCTTCAT	158	NM_001109986.1
P62	GGAAGCTGAAACATGGGCAC	CCAAGGGTCCACCTGAACAA	183	NM_181550.2
ATF6	AAGTGAAGAACCATTACTTTATATC	TTTCTGCTGGCTATTTGT	157	NM_001107196.1
BIP	AACCAAGGATGCTGGCACTA	ATGACCCGCTGATCAAAGTC	240	NM_013083.2
XBP1	TTACGAGAGAAAACTCATGGGC	GGGTCCAACTTGTCCAGAATGC	289	NM_001004210.2
PI3K	CCCTGCCCCATTTCATCCTT	TGTTGTTGCCCCAGACATGA	162	NM_053481.2
GAPDH	GGCACAGTCAAGGCTGAGAATG	ATGGTGGTGAAGACGCCAGTA	143	NM_017008.4

### Histopathological assessment of the sciatic nerve

Samples of the sciatic nerve were gathered and subsequently conserved in a solution of buffered neutral formalin, with a concentration of 10%, for 48 h. Next, dehydration occurred by raising the alcohol content of the samples. After being washed in xylene, they were embedded in paraffin. The tissue blocks were preserved in paraffin and subsequently sliced into 5 µm thick sections using a microtome (Leica RM 2155, England). The sections were dewaxed and stained with hematoxylin and eosin (H&E) [47].

### Immunohistochemical examination

The tissue sections, with a thickness of 5 μm and deparaffinization, were immersed in a solution containing 3% hydrogen peroxide (H_2_O_2_) for 30 min. The samples were then re-incubated at 37 °C for an additional hour. For this phase, we followed the manufacturer’s instructions to treat the slices with anti-Beclin 1 (MA5-15,825; Invitrogen, USA, 1:200), anti-LC3 (ab192890; Abcam, Cambridge, UK, 1:2000), and anti-p62 (ab91526; Abcam, Cambridge, UK, 1:1000). Following a thorough wash in PBS, the sections underwent treatment using a secondary antibody and the HRP Envision kit (DAKO) for 20 min. Once the slices were cleaned thoroughly with PBS, they were incubated with diaminobenzidine for 10 min. Afterward, the samples underwent a process of dehydration, followed by washing with xylene. Subsequently, they were counterstained with hematoxylin and placed under a cover slip for further examination using a microscope. The analysis was completed using the technique adopted by Elsayed et al. [48]. A set of seven non-overlapping separate fields was selected randomly. Selected areas were then investigated to determine the average percentage of tissue stained positive for Beclin 1, LC3, and p62 using immunohistochemistry for each tissue slice within a sample. Tissue slices were imaged with Full HD microscopic imaging equipment (Leica Microsystems Ltd.; Wetzlar, Germany) and analyzed with Leica Application software version 3.7.5.

### Statistical analysis

The data was analyzed using the GraphPad Prism software, specifically version 10.0.1, developed in San Diego, CA, USA. A one-way ANOVA was employed to examine the data, followed by the Tukey–Kramer test. The significance level of 0.05 was chosen as the threshold for determining statistical significance.

## Results

### Characterization of Chr-PLs

**Fig. 1:**
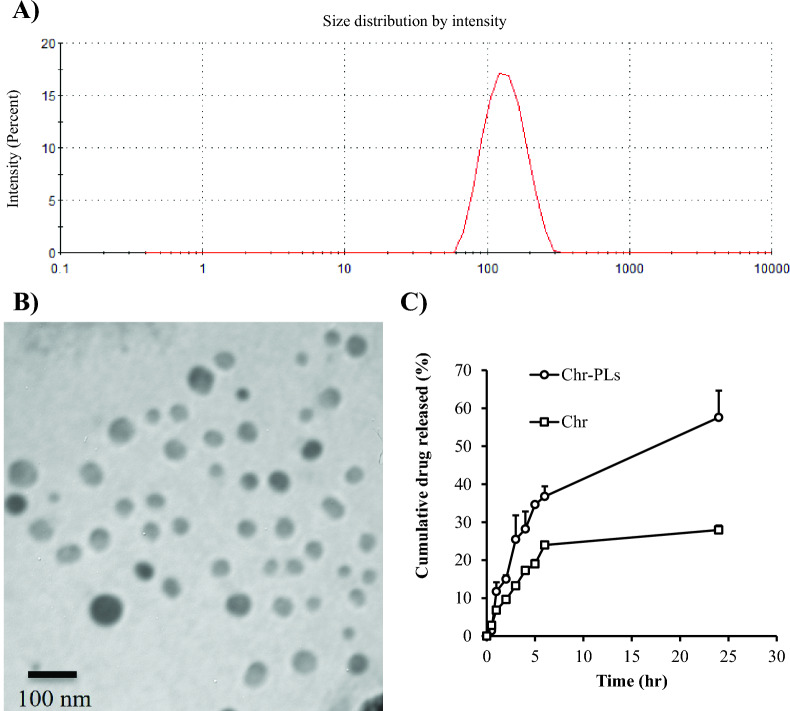
Characterization of Chr-PLs formulation. Chr-PLs were characterized for particle size diameter (**A**), TEM (**B**), and in vitro drug release (**C**). The preparation of Chr-PLs was accomplished using the ethanol injection approach. The spherical shape and nano-size dimensions of Chr-PLs were confirmed by its characterization. Drug release was found to be improved after formulation into nanoliposomes, according to in vitro release measurements. In Figure **C**, squares indicate Chr-PLs and circles indicate Chr

Chr-PLs were successfully fabricated using an ethanol injection methodology and evaluated for their particle size diameter, polydispersity score, zeta potential, TEM, and in vitro drug release. Within the nanoscale range, the average particle diameter of Chr-PLs was 134.6 ± 21.45 nm detected by Zetasizer nano (Malvern, UK) (Fig. 1A). Liposomal particles were also more evenly distributed, as seen by their polydispersity score of 0.275 ± 0.073. Furthermore, liposomal vesicles exhibit a slightly negative surface charge, measuring − 21.1 ± 1.72 mV. Images obtained by transmission electron microscopy (Jeol, Tokyo, Japan) showed that the Chr-PLs were nearly spherical, and the average particle diameter was like that found using Zetasizer nano (Fig. 1B). The liposomal vesicular systems also showed improved chrysin trapping capability, with an efficiency of 90.48 ± 7.75%. In vitro release assay demonstrates the cumulative release rate of Chr-PLs, as shown in Fig. 1C. Within the first six hours, 36.7% of preloaded Chr were released; after that, after 24 h, this percentage rose steadily to over 57.6%. By contrast, the Chr-free drug only released 23.98% of the loaded amount after 6 h, slightly increasing to 27.95% after 24 h.

### Chr-PLs enhance body weight index and serum level of acetylcholinesterase in alloxan-induced diabetes in rats

**Fig. 2:**
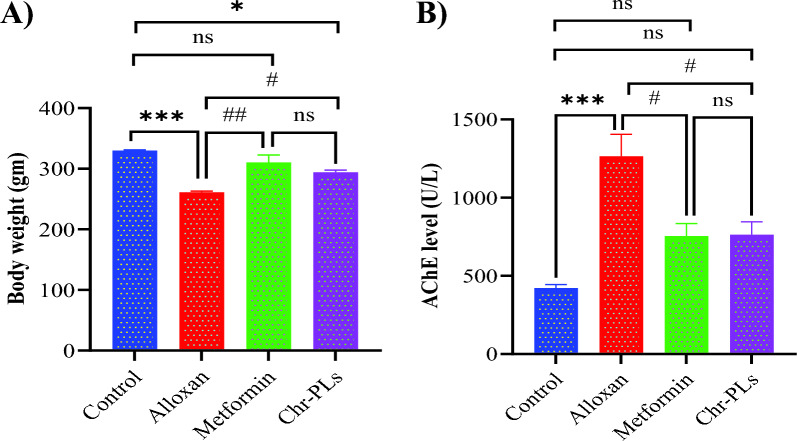
Influence of Chr-PLs administration on the body weight and serum AChE level in alloxan-induced diabetes in rats. The body weight (**A**) and serum AChE level (**B**) were measured for the different groups. ***P < 0.001, *P < 0.05 *vs.* control group. ^##^P < 0.01, ^#^P < 0.05 *vs.* alloxan group, ns indicates nonsignificant. Data are presented as the mean ± SEM (n = 10)

Next, we looked at how alloxan-induced diabetes in rats affected body weight and acetylcholinesterase levels to determine the impact of Chr-PLs. Alloxan markedly decreased body weight by 20.9% and elevated acetylcholinesterase levels by 199% compared to the control group. Diabetic rats’ body weight was significantly increased by 18.9%, and serum acetylcholinesterase was significantly lowered by 40% after injecting Chr-PLs with Alloxan (Fig. 2A and B). Results for the metformin group were comparable to those for the Chr-PLs group.

### Chr-PLs enhance blood levels of FBG and insulin, and the scores of HOMA-IR and HOMA-β in alloxan-induced diabetes in rats

**Fig. 3:**
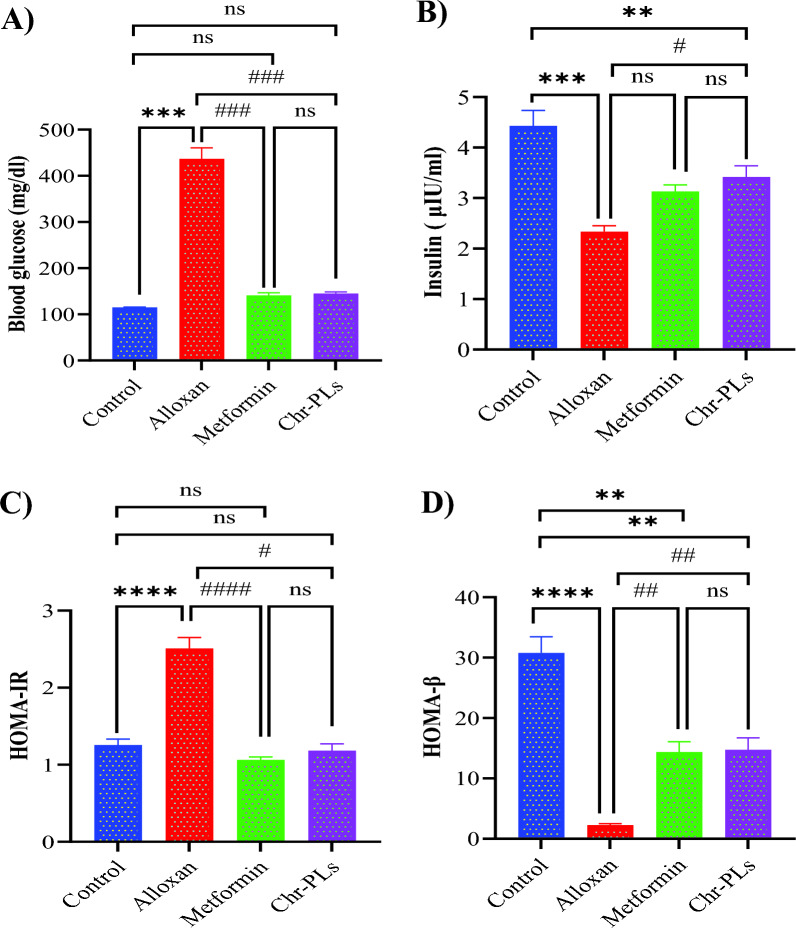
Influence of Chr-PLs administration on the levels of FBG, insulin, HOMA-IR, and HOMA-β in alloxan-induced diabetes in rats. The levels of FBG (**A**) and insulin (**B**) in blood and the scores for HOMA-IR (**C**) and HOMA-β (**D**) were measured for the different groups. ****P < 0.0001, ***P < 0.001, **P < 0.01 *vs.* control group. ^####^P < 0.0001, ^###^P < 0.001, ^##^P < 0.01, ^#^P < 0.05 *vs.* alloxan group, ns indicates nonsignificant. Data are presented as the mean ± SEM (n = 10)

The antidiabetic effects of Chr-PLs were also evaluated by measuring blood levels of FBG and insulin and the scores of HOMA-IR and HOMA-β. In contrast to the control group, it was revealed that after receiving Alloxan, FBG increased by 279%, and HOMA-IR increased by 99.7%. In comparison, plasma insulin levels decreased by 47.3%, and HOMA-β levels rose by 92.6%. FBG and HOMA-IR were reduced by 67.7% and 57.6%, respectively, when Alloxan and Chr-PLs were administered concurrently. In contrast, plasma insulin and HOMA-β significantly increased by 31.4% and 532%, respectively, compared to the alloxan group. Similar outcomes were seen in the metformin-treated group as in the Chr-PLs group (Fig. 3A–D).

### Chr-PLs downregulate mRNA expression levels of ER stress genes in the sciatic nerve in alloxan-induced diabetic rats

**Fig. 4:**
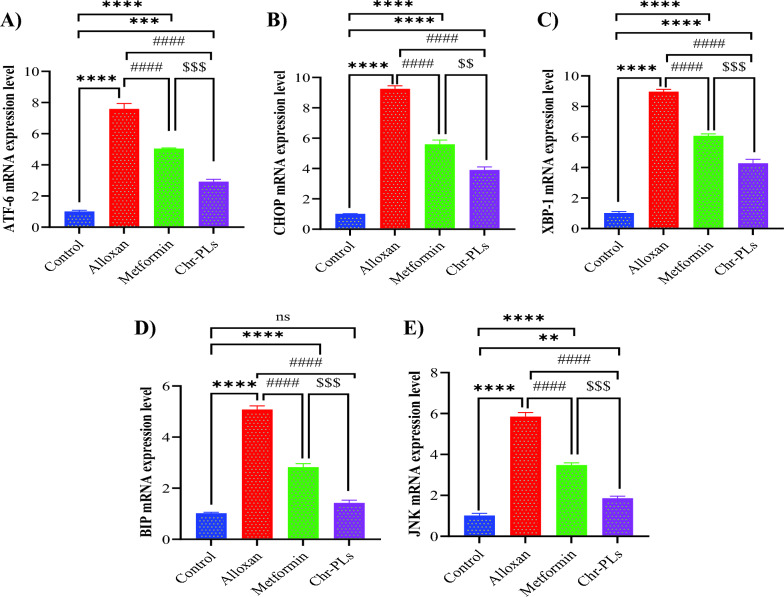
Influence of Chr-PLs administration on the mRNA expression of ER stress markers in the sciatic nerve in alloxan-induced diabetes in rats. The mRNA levels of ER stress markers, including ATF6 (**A**), CHOP (**B**), XBP-1 (**C**), BIP (**D**), and JNK (**E**) were measured for the different groups. ****P < 0.0001, ***P < 0.001, **P < 0.01 vs. control group. ^####^P < 0.0001*vs.* alloxan group. ^$$$^P < 0.001, ^$$^P < 0.01 vs. metformin group, ns indicates nonsignificant. Data are presented as the mean ± SEM (n = 10)

Subsequently, the influence of Chr-PLs on the mRNA expression levels of ER stress genes (ATF6 signaling pathway) was examined in an alloxan-induced DN model. After administering Alloxan, mRNA expression levels of all examined ER genes were markedly increased. The expression levels of activating transcription factor 6 (ATF6), C/EBP homologous protein (CHOP), X-box-binding protein 1 (XBP-1), binding immunoglobulin protein (BIP), and Jun N-terminal kinase (JNK) were increased by 650%, 817%, 782%, 337%, and 474%, respectively, in the sciatic nerve. However, compared to the alloxan group, Chr-PLs resulted in a considerable suppression of mRNA expression levels of ATF6, CHOP, XBP-1, BIP, and JNK by 33%, 39.5%, 32.2%, 44.4%, and 40.4%, respectively. The Chr-PLs group showed better results than the metformin-treated group (Fig. 4A–E).

### Chr-PLs enhance mRNA expression levels of autophagy-dependent markers in the sciatic nerve in alloxan-injected rats

**Fig. 5:**
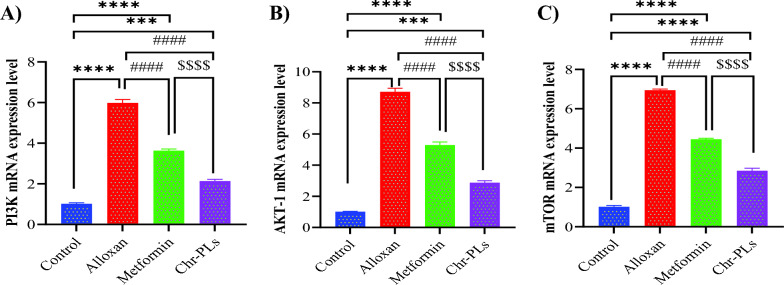
Influence of Chr-PLs administration on the mRNA expression of autophagy-dependent markers in the sciatic nerve in alloxan-induced diabetes in rats. The mRNA levels of ER stress markers, including PI3K, Akt, and mTOR, were measured for the different groups. ****P < 0.0001, ***P < 0.001 *vs.* control group. ^####^P < 0.0001 *vs.* alloxan group. ^$$$$^P < 0.0001 *vs.* metformin group. Data are presented as the mean ± SEM (n = 10)

The impact of Chr-PLs administration on the expression levels of autophagy-dependent genes was also investigated. The influence of Chr-PLs treatment on PI3K/Akt1/mTOR pathway genes in the sciatic nerve in alloxan-injected rats was evaluated. Alloxan administration resulted in a notable rise in the expression levels of phosphoinositide 3-kinase (PI3K), Akt isoform 1 (Akt1), and Mammalian target of rapamycin (mTOR) by 486%, 760%, and 581%, respectively, in the sciatic nerve in contrast to the control group. The administration of Chr-PLs resulted in a noteworthy reduction of 39.2%, 39%, and 35.9% in the mRNA expression levels of PI3K, Akt1, and mTOR, respectively, compared to the group induced with Alloxan (Fig. 5A–C). Furthermore, as shown in Fig. 5A–C, the results obtained by the Chr-PLs group were superior to those obtained by the metformin group.

**Fig. 6:**
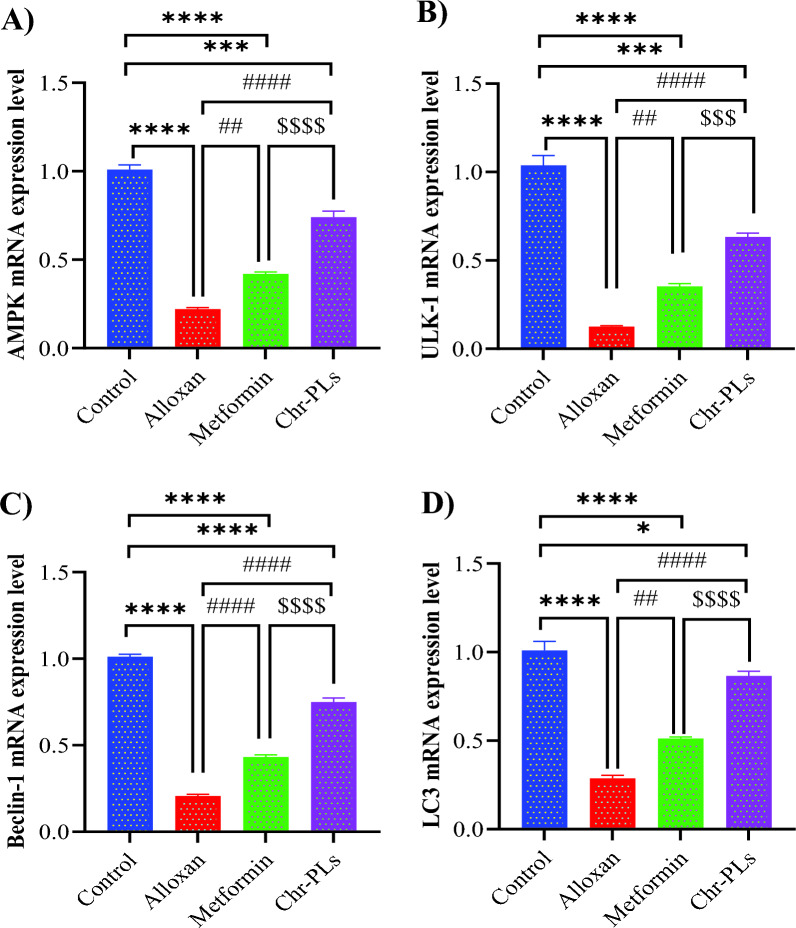
Influence of Chr-PLs administration on the mRNA expression of autophagy-dependent markers in the sciatic nerve of alloxan-induced diabetes in rats. The mRNA levels of AMPK (**A**), ULK-1 (**B**), beclin 1 (**C**), and LC3 (**D**) were measured for the different groups. Data are presented as the mean ± SEM (n = 10). ****P < 0.0001, ***P < 0.001, *P < 0.05 *vs.* control group. ^####^P < 0.0001, ^##^P < 0.01*vs.* alloxan group. ^$$$$^P < 0.0001, ^$$$^P < 0.001 vs. metformin group

The mRNA levels of AMP-activated protein kinase (AMPK), unc-51 like autophagy activating kinase 1 (ULK-1), bcl-2 interacting protein 1 (beclin 1), and microtubule-associated proteins 1A/1B light chain 3B (LC3) in the sciatic nerve of alloxan-injected rats were considerably lower than those in the control group by 78%, 87.8%, 79.5%, and 71.5%, respectively. However, administering Chr-PLs to alloxan-induced diabetic rats reversed the downregulation of autophagy-dependent gene expression. Sciatic nerve mRNA levels for AMPK, ULK-1, beclin 1, and LC3 were all significantly increased in the Chr-PLs group compared to the alloxan group by 90.3%, 181%, 109%, and 78%, respectively. The outcomes for Chr-PLs were superior to those of the metformin group (Fig. 6A–D).

### Chr-PLs modulate miRNA expression levels of miR-301a-5p and miR-30e-5p in the sciatic nerve in alloxan-induced diabetic rats

**Fig. 7:**
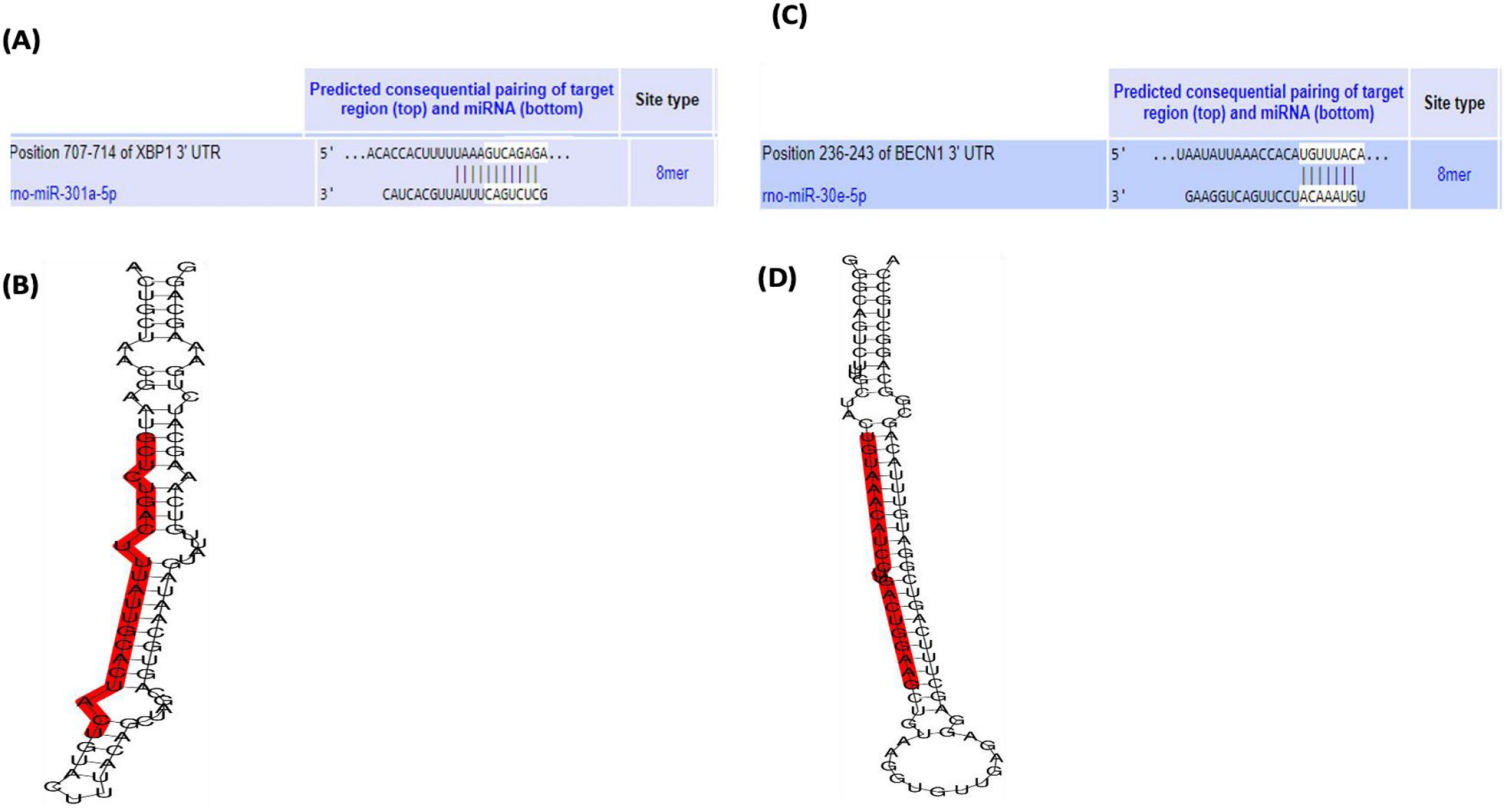
location of the 3′ UTR region for miRNA binding to the targeted mRNA, (**A**, **B**) miR-301a-5p, (**C**, **D**) miR-30e-5p

**Fig. 8:**
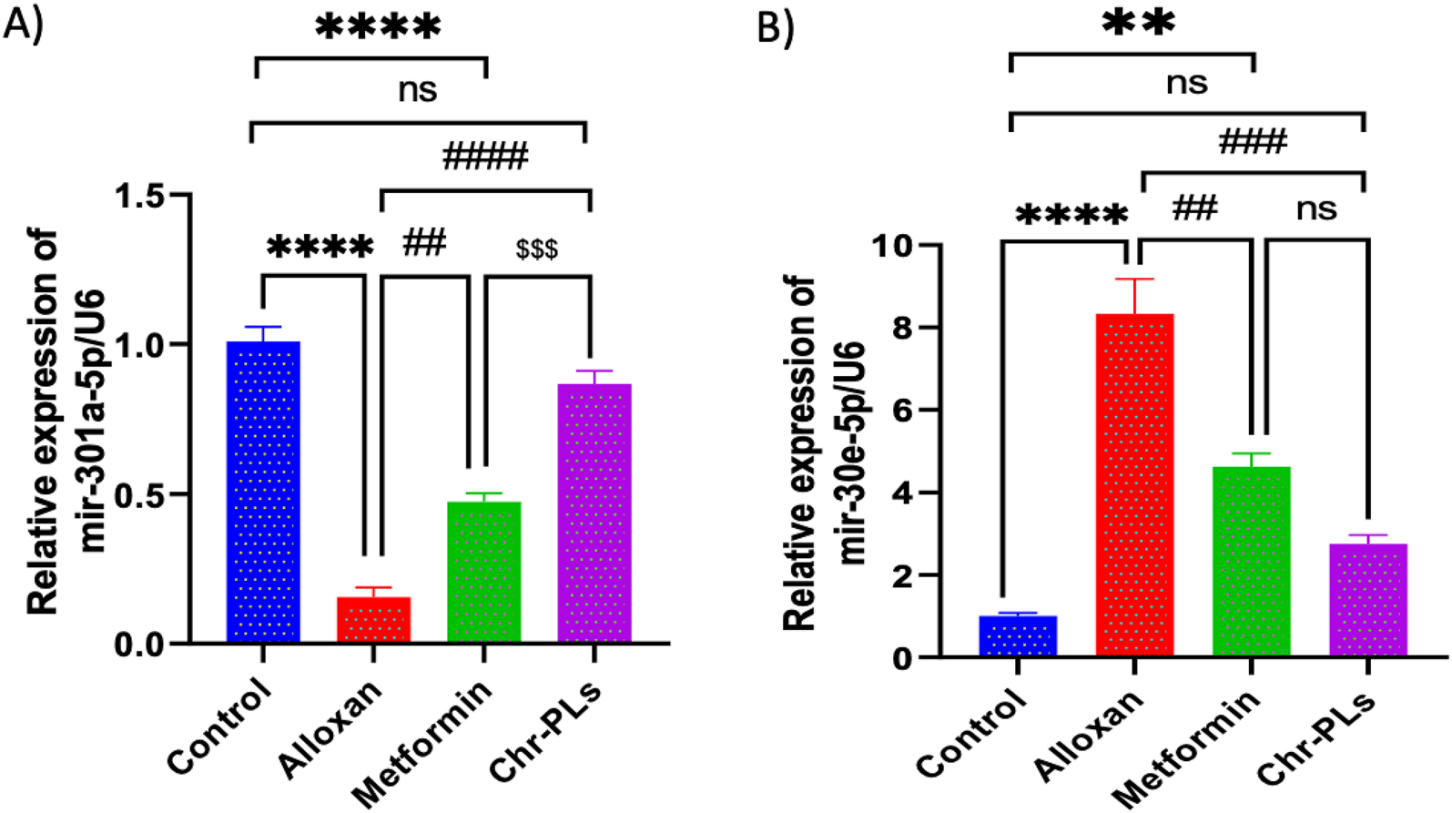
Influence of Chr-PLs administration on the expression of miR-301a-5p (**A**), and miR-30e-5p (**B**) in the sciatic nerve of alloxan-induced diabetes in rats. ****P < 0.0001, **P < 0.01 *vs.* control group. ^####^P < 0.0001, ^###^P < 0.001, ^##^P < 0.01*vs.* alloxan group. ^$$$^P < 0.001 vs. metformin group

The location of the 3′ UTR region for miRNA binding to the targeted mRNA is shown in (Fig. 7). Theinfluence of Chr-PLs treatment on miRNA expression levels of miR-301a-5p and miR-30e-5p in thesciatic nerve in alloxan-injected rats was evaluated. The influence of Chr-PLs treatment on miRNA expression levels of miR*-*301a*-*5p and miR*-*30e*-*5p in the sciatic nerve in alloxan-injected rats was evaluated. Alloxan administration resulted in a notable drop in the expression levels of miR*-*301a*-*5p by 84% and a marked rise in the expression levels of miR*-*30e*-*5p by 732% in the sciatic nerve compared to the control group. The administration of Chr-PLs resulted in a noteworthy upregulation in the miR*-*301a*-*5p expression levels by 513% and downregulation in the miR*-*301a*-*5p expression levels by 65% compared to the group induced with Alloxan (Fig. 8A, B). Furthermore, as shown in Fig. 8, the results obtained by the Chr-PLs group were superior to those obtained by the metformin group.

### Chr-PLs restore histological changes in the sciatic nerve in alloxan-injected rats

**Fig. 9:**
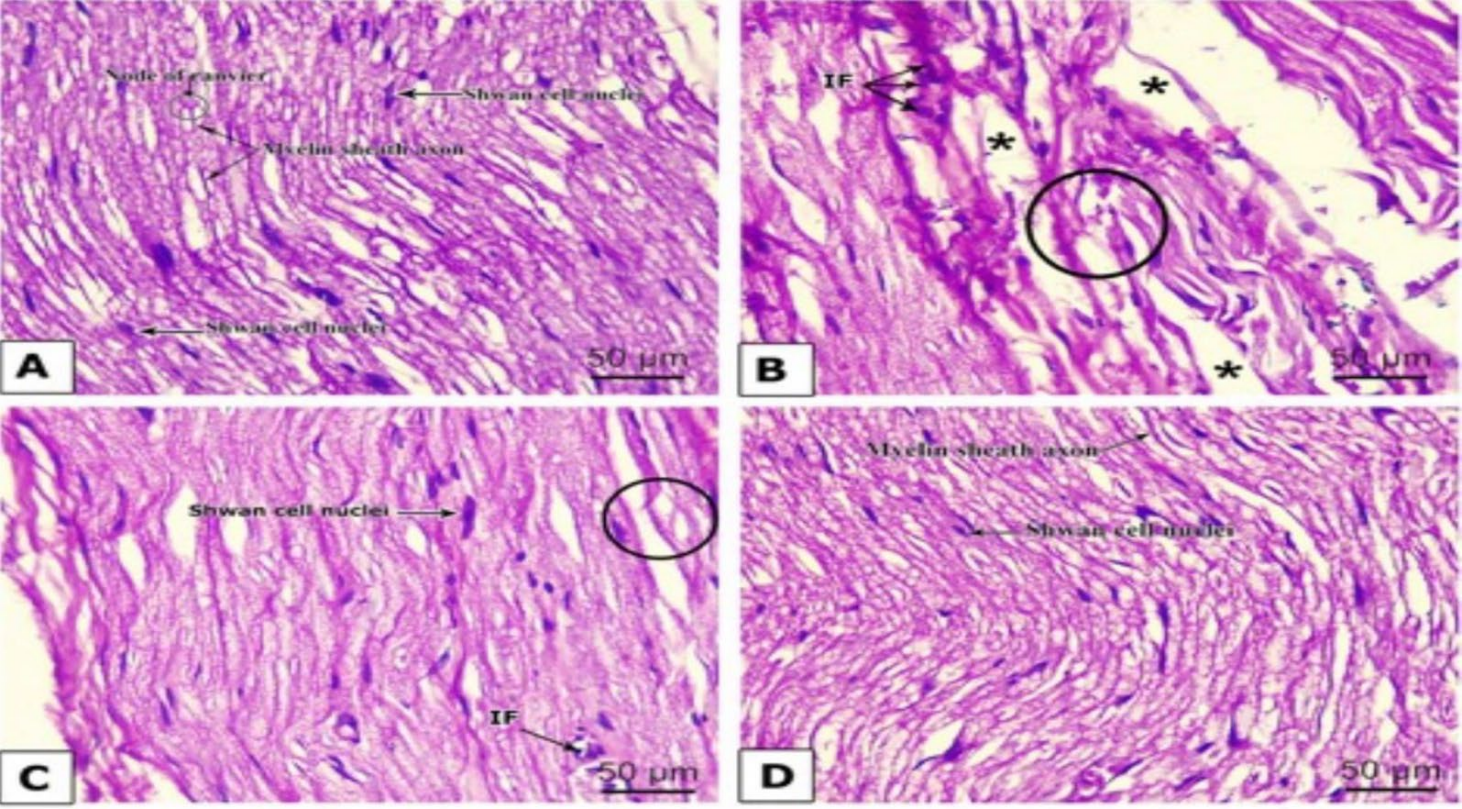
Influence of Chr-PLs administration on histopathological changes in the sciatic nerve of alloxan-induced diabetes in rats. Histopathological examination of the sciatic nerve of control (**A**), Alloxan (**B**), metformin (**C**), and Chr-PLs (**D**). (o) indicates myelin sheath degeneration; (*) indicates separation between the nerve fibers; and (IF) indicates mononuclear infiltrating cells. Schwann cell nuclei, myelin sheath axons, and node of Ranvier can be observed

Longitudinal sections of the peripheral nerve bundles of the control group stained with hematoxylin and eosin revealed many well-organized myelinated intact axons. The nodes of Ranvier are visible between adjacent internodes of the myelin sheath. The nuclei of Schwann cells can be seen through the vacuolated myelin sheaths (Fig. 9A). Contrarily, myelin sheath degeneration and nerve fiber separation were observed in multiple stained nerve slices taken from alloxan-treated rats. Cellular infiltrations could be noticed (Fig. 9B). Most myelin sheath integrity was restored with normal Schwann cell nuclei in metformin-treated diabetic rat tissue slices. However, there was still some evidence of nerve fiber disconnection and cellular infiltration (Fig. 9C). Sciatic nerves of Chr- PLs-treated diabetic rats appear similar to those of the control group, which is an intriguing finding (Fig. 9D).

### Immunohistochemical examination

**Fig. 10:**
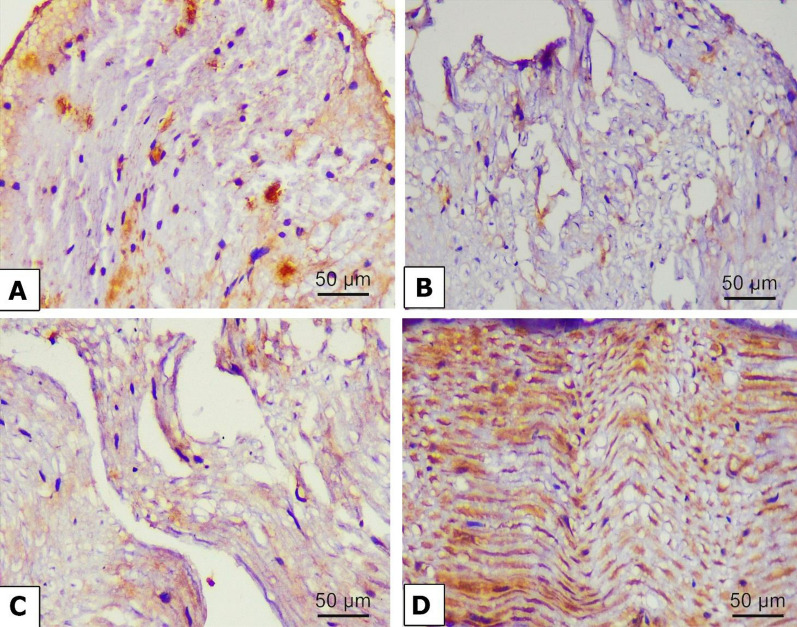
Representative microscopic images displaying the levels of Beclin 1 immuno-expression in the sciatic nerve fibers of alloxan-induced diabetes in rats. Expression of Beclin 1 in the sciatic nerve of control (**A**), Alloxan (**B**), metformin (**C**), and Chr-PLs (**D**). Positive staining was indicated by a brown-yellow color (Scale bar; 50 µm and magnification power; 400 ×)

**Fig. 11:**
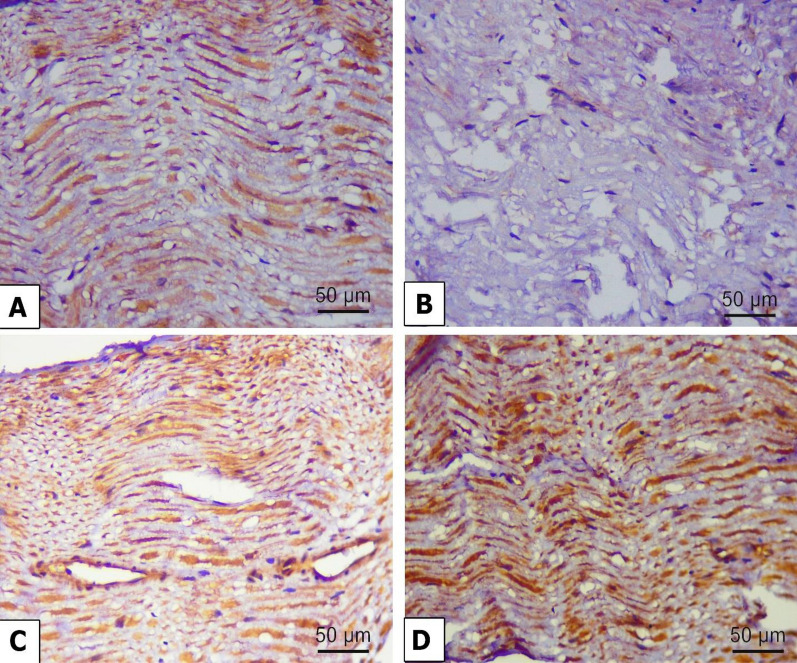
Representative microscopic images displaying the levels of LC3 immuno-expression in the sciatic nerve fibers of alloxan-induced diabetes in rats. Expression of LC3 in the sciatic nerve of control (**A**), Alloxan (**B**), metformin (**C**), and Chr-PLs (**D**). Positive staining was indicated by a brown-yellow color (Scale bar; 50 µm and magnification power; 400 ×)

**Fig. 12:**
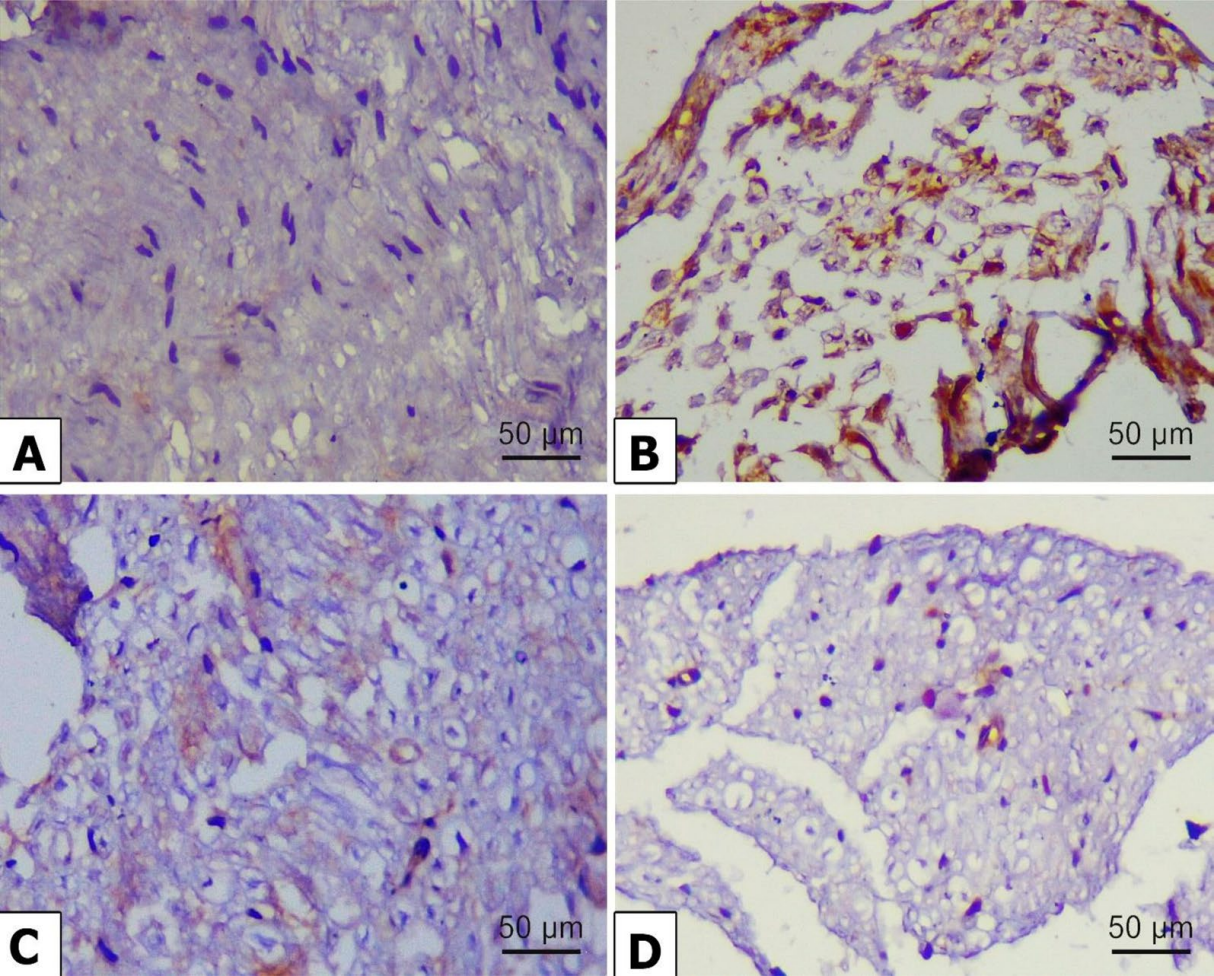
Representative microscopic images displaying the levels of P62 immuno-expression in the sciatic nerve fibers of alloxan-induced diabetes in rats. Expression of P62 in the sciatic nerve of control (**A**), Alloxan (**B**), metformin (**C**), and Chr-PLs (**D**). Positive staining was indicated by a brown-yellow color (Scale bar; 50 µm and magnification power; 400 ×)

The changes in autophagy markers in the nerve fibers were estimated by measuring the immunohistochemical expression of beclin 1, LC3, and p62. Beclin 1 (Fig. 10A) and LC3 (Fig. 11A) exhibited positive expressions in the nerve fibers of the control group. However, beclin 1 (Fig. 10B) and LC3 (Fig. 11B) showed weak expression in the alloxan-treated group. However, P62 expression was poorly expressed in the control group (Fig. 12A) and strongly expressed in the alloxan group (Fig. 12B). The findings imply that hindering the process of autophagy could potentially enhance the expression of p62. Chr-PLs treatment resulted in increased expression of beclin 1 (Fig.10D) and LC3 (Fig. 11D) and decreased expression of p62 (Fig. 12D). The results obtained via the administration of Chr-PLs were comparable to those of the anti-hyperglycemic drug metformin (see Figs. 10-12).

## Discussion

Insufficient insulin synthesis, action, or both characterize the metabolic disorder known as diabetes. This results in persistently high blood sugar levels and difficulty metabolizing carbohydrates, proteins, and fats [49]. Nephropathy and neurologic consequences are included in this illness [50]. In this study, our findings demonstrate the significant involvement of the ER stress and autophagy signaling pathway in the capacity of Chr-PLs to safeguard against advancing diabetic neuropathy. Diabetes was produced in rats via the administration of Alloxan. Insufficient insulin production and high blood sugar levels result from the preferential uptake of the oxygenated pyrimidine alloxan by pancreatic β-cells [51]. The biopharmaceutical classification system (BCS) assigns Chr a class II classification, indicating its low water solubility and restricted bioavailability [52]. One way to overcome this challenge has been to suggest incorporating chrysin into suitable delivery systems. Chr was thus included in PEGylated liposomal systems to improve its pharmacokinetic profile and offer a longer half-life [53]. The effective encapsulation of chrysin into liposomal systems improved its biological activity against diabetes and allowed for better formulation into aqueous preparations.

The mitigation of alloxan-induced diabetic neuropathy effects may be influenced by the different routes by which metformin and Chr-PLs are administered. Metformin is typically administered orally. The administration pathway can influence the bioavailability and pharmacokinetics of these drugs. Metformin's widespread use faces issues like low bioavailability, high dose, frequent dosage, gastrointestinal side effects, and poor absorption due to its cationic biguanide structure [54]. Chrysin liposome, on the other hand, may have higher bioavailability when administered intraperitoneal due to bypassing the gastrointestinal tract. Ahmed, et al. [55] found that gold nanoparticles administration by intraperitoneal route resulted in a more significant anti-fibrotic effect against hepatic Schistosoma mansoni infection than oral administrated nanoparticles. Therefore, chrysin liposome administered intraperitoneal may have a more potent effect on the target tissues than orally administered metformin and this serves a part of our study design which mainly focuses on comparing the potential effect of intraperitoneal injected chrysin liposome comparing standard orally administrated metformin already used in market.

Metformin primarily acts by reducing hepatic glucose production and improving insulin sensitivity. The antidiabetic effect of metformin is primarily achieved by inhibiting hepatic gluconeogenesis [56]. Metformin is widely recognized for targeting hepatic mitochondria and inhibiting mitochondrial respiratory chain complex I, which is reversible and weak [57]. Furthermore, the slight rise in intracellular AMP levels triggered by metformin inhibits AMP-regulated hepatic gluconeogenesis-related enzymes, including fructose-1,6-bisphosphatase and adenylate cyclase. This, in turn, results in a reduction in the production of glucose in the liver and activates the cellular energy sensor AMPK [58, 59]. Chrysin liposome exerts antioxidant, anti-inflammatory, and antiapoptotic effects, potentially modulating various cellular pathways. It is a flavonoid with a diphenylpropane skeleton system that exerts significant anti-oxidant and anti-inflammatory properties due to its hydroxyl substituent position. It upregulates the transcription factor Nrf2, a crucial transcription factor responsible for anti-oxidant effects [60]. Chrysin's anti-inflammatory properties have been demonstrated to be neuroprotective after cerebral ischemia by modulating estrogen receptors [61]. It also mitigates dopamine depletion and safeguards against the neurodegeneration of dopaminergic neurons in the brain [62]. It can limit neuroinflammation by modulating JNK and NF-κB expression and attenuating PI3K/AKT/mTOR and NLRP3 inflammasome pathways [63, 64]. Chrysin-loaded magnetic PEGylated silica nanospheres in animals reduce Aβ-induced memory impairment by reducing lipid peroxidation levels and increasing antioxidant molecules, thereby promoting neuroprotection [65].

The effective encapsulation of chrysin into liposomal systems improved its biological activity against diabetes and allowed for better formulation into aqueous preparations. One extremely prevalent feature of DM is abnormal body weight loss. Based on our findings, Alloxan causes a considerable decrease in body weight levels. These results aligned with those obtained by Tasnin et al. [66], who found a reduction in the body weight of diabetic rats. However, treatment with Chr-PLs significantly improved weight loss. Diabetes-related cognitive deficits, memory loss, and neurophysiological abnormalities were found to include AChE [67]. Consistent with the findings of Okesola et al. [68], the results demonstrated an elevation of serum AChE in alloxan-treated rats. Treatment with Chr-PLs, on the other hand, considerably reduced serum AChE levels. Streptozocin-induced diabetic foot ulcers in rats were found to be improved by chrysin treatment, as reported by Liu et al. [69]. Here, the administration of Chr-PLs to diabetic rats suppressed AChE activity, which could lessen acetylcholine hydrolysis and alleviate neuronal damage. The most notable change in diabetic sciatic nerves, as revealed by histologic examination, is the numerous losses of myelin sheath axons, accompanied by dissociation of nerve fibers and cellular infiltration. The neurodegenerative alterations in the sciatic nerve of diabetic rats were, however, alleviated by treatment with Chr-PLs. The results matched those of Pathak et al. [70], who showed that alloxan administration displayed abnormal sciatic nerve fibers and substantial axonal edema while decreasing axonal degeneration in the chrysin nanoemulsion group. These results indicate the regenerative characteristics of Chr-PLs.

Diabetes-induced rats exhibited a reduction in blood insulin and HOMA-β, whereas the FBG and HOMA-IR increased. The same findings were found by Gharib et al. [67]. In diabetic rats, an increase in the HOMA-IR score and a considerable fall in fasting blood insulin levels are indicative of an insulin-resistant state. Serum insulin and HOMA-β levels were significantly increased in Chr-PLs-treated diabetic rats, whereas FBG and HOMA-IR were markedly decreased. These results were coordinated with the results obtained by Salama et al. [50], and Kim et al. [71]. Increased peripheral uptake of glucose, suppression of hepatic glucose production [71], and improved insulin sensitivity may all contribute to chrysin’s powerful hypoglycemic impact.

When several stressors disrupt the ER’s regular activity, the resulting accumulation of unfolded and misfolded proteins in the ER’s lumen is known as ER stress [7]. Protein kinase RNA-like ER kinase (PERK), activating transcription factor 6 (ATF6), and inositol-requiring enzyme 1 (IRE1) are the three regulatory mechanisms that control UPR. They regulate various cellular processes, such as autophagy, apoptosis, the antioxidant response, and inflammation [72]. Eukaryotic initiation factor-2 (eIF2) is phosphorylated by PERK during an ER stress response, activating CCAAT-enhancer-binding protein homologous protein (CHOP) [73]. Dissociation of the chaperone protein BiP from the initiation factor IRE1 triggers trans-autophosphorylation and the processing of unspliced, inactive XBP1 mRNA (uXBP1) into the spliced, active sXBP1, which stimulates transcription of specific target genes [74]. The formation of vacuoles or autophagosomes requires IRE1-mediated activation of JNK. The adaptive responses produced by ER autophagy offset the damaging consequences of ER stress, prolong cell viability, and delay apoptosis. However, prolonged high blood sugar levels interfere with the autophagy mechanism, leading to cell death [75]. Autophagy indicators have involved Beclin 1, LC3A/B, and p62 expression levels [74]. Phosphorylation of the autophagy-related protein UNC-51-like kinase 1 (ULK1) by the AMP-activated protein kinase (AMPK) stimulates autophagy [76]. The PI3K/Akt/mTOR pathway inhibition activates autophagy and suppresses cancer cell proliferation [77].

The present investigation observed an increase in the levels of markers associated with ER stress and autophagy in rats with diabetes. These markers included ATF6, CHOP, XBP-1, BIP, JNK, PI3K, Akt, mTOR and p62. However, there was marked downregulation in the AMPK, ULK-1, beclin 1, and LC3 levels. Our results were confirmed by a decrease in the expression levels of miR*-*301a as a target for XBP-1 which augments ER stress response and a rise in the expression levels of miR*-*30e*-*5p as a target for Beclin1 which inhibits autophagy [69]. Both defective UPR and impaired autophagy have been associated with the onset of many forms of neurodegeneration [78]. Methamphetamine, for instance, can cause glial cells to experience ER stress by activating the UPR and leading to elevated PERK phosphorylation, ATF6 expression, and p-IRE1 enzyme activity [79].

Additionally, BiP, CHOP, and XBP-1 mRNA expression levels are raised [80]. Insulin resistance induces overexpression of ER stress indicators such as p-eIF2, ATF4, CHOP, sXBP1, p-IRE1, and p-ASK1 at the mRNA or protein level, causing neuronal cell death in neuroblastoma cells [81]. Autophagy was also impaired in a mouse model of neuropathic pain, as evidenced by the downregulation of LC3 and Beclin 1 and increased p62 protein expression following spinal nerve ligation [82]. ER stress was significantly reduced, and autophagy was enhanced after the administration of Chr-PLs. This finding agreed with Kang et al. [83], who treated glucose-stimulated human RPE cells with chrysin and observed that ER stress-related sensors such as BiP, ATF6, and the IRE1 pathway were less induced. In a study using a D-galactose model of aging in mice, Salama et al. [50] found that chrysin prevented neurodegeneration, boosted mitochondrial autophagy and biogenesis, and reduced oxidative stress and neuroinflammation. The increased ER stress response and the suppression of autophagy in the alloxan model of DN may be the outcomes of hyperglycemia’s enhancement of oxidative stress.

## Conclusion

Ultimately, our investigation shows that Chr-PLs protect rats from diabetic neuropathy produced by Alloxan. Attenuation of ER stress and induction of autophagy regulation are probably one of the mechanisms by which these effects work. The underlying processes and therapeutic potential of Chr-PLs in treating diabetic neuropathy require further research.

## Data Availability

The datasets utilized and analyzed in the ongoing investigation can be made available by the author responsible for correspondence.

## Abbreviations

AChE: Acetylcholinesterase
Akt1: Akt isoform 1
AMPK: AMP-activated protein kinase
ATF6: Activating transcription factor 6
Beclin 1: Bcl-2 interacting protein 1
BIP: Binding immunoglobulin protein
Chr: Chrysin
Chr-PLs: Chrysin PEGylated liposomes
CHOP: C/EBP homologous protein
DN: Diabetic neuropathy
DM: Diabetes mellitus
ER: Endoplasmic reticulum
FBG: Fasting blood glucose
HOMA-IR: Homeostasis model assessment of insulin resistance
HOMA-β: The Homeostasis Model Assessment of Beta Cell Function
JNK: Jun N-terminal kinase
LC3: Microtubule-associated proteins 1A/1B light chain 3B
mTOR: Mammalian target of rapamycin
PEG_4000_: Polyethylene glycol4000
PI3K: Phosphoinositide 3-kinase
TEM: Transmission electron microscopy
ULK-1: Unc-51 like autophagy activating kinase 1
UPR: Unfolded protein response
XBP-1: X-box-binding protein 1
